# Enhanced Growth Inhibition of Osteosarcoma by Cytotoxic Polymerized Liposomal Nanoparticles Targeting the Alcam Cell Surface Receptor

**DOI:** 10.1155/2012/126906

**Published:** 2012-09-11

**Authors:** Noah Federman, Jason Chan, Jon O. Nagy, Elliot M. Landaw, Katelyn McCabe, Anna M. Wu, Timothy Triche, HyungGyoo Kang, Bin Liu, James D. Marks, Christopher T. Denny

**Affiliations:** ^1^Division of Pediatric Hematology/Oncology, Department of Pediatrics, Mattel Children's Hospital and Gwynne Hazen Cherry Memorial Laboratories, UCLA Jonsson Comprehensive Cancer Center, Los Angeles, CA 90095-1781, USA; ^2^NanoValent Pharmaceuticals Inc., Bozeman, MT 59718-4012, USA; ^3^Department of Biomathematics, David Geffen School of Medicine, UCLA, Los Angeles, CA 90095-1766, USA; ^4^Department of Molecular and Medical Pharmacology, Crump Institute for Molecular Imaging, UCLA, Los Angeles, CA 90095-1770, USA; ^5^Department of Pathology and Laboratory Medicine, Children's Hospital Los Angeles, University of Southern California, Los Angeles, CA 90027, USA; ^6^Department of Anesthesia and Perioperative Cave, Helen Diller Family Comprehensive Cancer Center, University of California, San Francisco, CA 94110, USA

## Abstract

Osteosarcoma is the most common primary malignancy of bone in children, adolescents, and adults. Despite extensive surgery and adjuvant aggressive high-dose systemic chemotherapy with potentially severe bystander side effects, cure is attainable in about 70% of patients with localized disease and only 20%–30% of those patients with metastatic disease. Targeted therapies clearly are warranted in improving our treatment of this adolescent killer. However, a lack of osteosarcoma-associated/specific markers has hindered development of targeted therapeutics. We describe a novel osteosarcoma-associated cell surface antigen, ALCAM. We, then, create an engineered anti-ALCAM-hybrid polymerized liposomal nanoparticle immunoconjugate (*α*-AL-HPLN) to specifically target osteosarcoma cells and deliver a cytotoxic chemotherapeutic agent, doxorubicin. We have demonstrated that *α*-AL-HPLNs have significantly enhanced cytotoxicity over untargeted HPLNs and over a conventional liposomal doxorubicin formulation. In this way, *α*-AL-HPLNs are a promising new strategy to specifically deliver cytotoxic agents in osteosarcoma.

## 1. Introduction

Osteosarcoma is the most common primary malignant neoplasm of bone in children and adolescents and is characterized by unregulated proliferation of primitive osteoid-producing mesenchymal cells [1]. Prior to 1970, the prognosis for patients with osteosarcoma who were treated with surgery alone was a dismal 10%–20% overall survival. Though aggressive surgeries would render most patients grossly tumor-free, the vast majority would develop progressively fatal metastatic disease within two years. This suggested that at the time of their initial diagnosis clinically undetectable tumor had already spread to distant sites in most patients and that effective systemic anticancer therapy was needed [2].

The development of neoadjuvant cytotoxic chemotherapy regimens over the past three decades has dramatically improved the fate of osteosarcoma patients. The addition of multiagent regimens plus refinement in surgical resection has resulted in a 65%–75% long-term survival rate in patients presenting with localized disease [3]. While this is a substantial improvement, current multimodality therapy still has significant shortcomings. First, the outlook remains poor for patients with radiographically detectable metastases at diagnosis or for those in whom the cancer recurs. Second, while the currently utilized chemotherapy regimens are effective against osteosarcoma, they also wreak havoc on normal cells that can result in acute and potentially life-threatening complications. It is also now appreciated that exposure of pediatric cancer patients to cytotoxic chemotherapy can lead to secondary malignancies and other medical maladies, decades after their tumor has been eradicated [4].

 The long-sought goal of being able to preferentially deliver anticancer therapy to tumors while sparing normal cells could have a significant impact on the deficiencies of current osteosarcoma treatment regimens. In this regard, the use of nanoparticles as delivery vehicles appears promising. Liposomes, unilamellar vesicles composed of natural and/or synthetic lipids, have been a particularly intensively studied system [5]. The problem of containment versus controlled release of anti-cancer agents has been a challenge for liposomal drug delivery. On the one hand, liposomes need to be formulated to allow for efficient packaging of therapeutic agents and stable containment of drug in a normal extracellular environment. On the other hand, liposomes that have localized to tumors need to be able to release their payload in order to have a therapeutic effect. This latter attribute has been particularly difficult to program into standard liposome formulations [6, 7].

 Many nanoparticle anti-cancer targeting strategies require identification of a marker that is expressed on the surface of the tumor cell. In particular, tumor-associated molecules that are expressed at higher levels than in normal tissues are sought since nanoparticles coated with antibodies recognizing these markers can preferentially bind to tumor cells. Finally from a potential therapeutic delivery perspective, it is best when candidate tumor markers are internalized when bound by ligands or proteins at the cell surface [8]. By exploiting this interaction, targeted nanoparticles can deliver therapeutic payloads into tumor cells through receptor-mediated endocytosis.

 With these criteria in mind, the cell surface receptor activated leukocyte adhesion molecule (ALCAM, CD166) is an attractive candidate to target osteosarcoma. This glycoprotein is a member of the immunoglobulin superfamily and is thought to mediate important cell-cell interactions involved in cell migration, neurogenesis, hematopoiesis, and the immune response [9]. More recently, increased ALCAM expression has been linked to a variety of cancers including pancreatic, breast, prostate, and colorectal carcinomas and melanoma [10–12]. Furthermore, others have found that immunoliposomes coated with a recombinant anti-ALCAM monoclonal antibody were taken up by prostate cancer cell lines expressing this antigen [13]. 

 In this paper, we demonstrate that ALCAM is overexpressed in both osteosarcoma tumor-derived cell lines and primary biopsy specimens. We show that this cell surface molecule can be exploited to enhance binding and uptake of nanoparticles by osteosarcoma cells. We present a new polymerized liposome formulation consisting of a mixture of lipids with saturated and diacetylene containing acyl chains that when loaded with doxorubicin displays enhanced cytotoxicity to osteosarcoma cells. Finally, we find that coating these hybrid liposomes with recombinant anti-ALCAM antibody further improves cytotoxic killing of osteosarcoma cell lines.

## 2. Methods

### 2.1. Materials

Conventional and polymerized liposomal nanoparticles (PLNs and HPLNs) were obtained from NanoValent Pharmaceuticals, Inc. (Bozeman, MT, USA). The components comprising the conventional liposomes are L-*α*-phosphatidylcholine hydrogenated soy, (hydrogenated soy PC), cholesterol and 1,2-distearoyl-*sn*-glycero-3-phosphoethanolamine-N-[methoxy(polyethylene glycol)-2000] (m-PEG_2000_-DSPE), (Avanti Polar Lipids, Alabaster, AL, USA). The PLNs are comprised of (5′-hydroxy-3′-oxypentyl)-10-12-pentacosadiynamide (h-PEG_1_-PCDA), (5′-sulfo-3′-oxypentyl)-10-12-pentacosadiynamide (sulfo-PEG_1_-PCDA), N-[(methoxy(polyethylene glycol)-750]-10-12-pentacosadiynamide (m-PEG_750_-PCDA) and N-[(maleimide(polyethylene glycol)-1500]-10-12-pentacosadiynamide (mal-PEG_1500_-PCDA) (NanoValent Pharmaceuticals, Inc. Bozeman, MT, USA) and the HPLNs are comprised h-PEG_1_-PCDA, hydrogenated soy PC, m-PEG_2000_-DSPE, 1,2-distearoyl-*sn*-glycero-3-phosphoethanolamine-N-[maleimide(polyethylene glycol)-2000] (mal-PEG_2000_-DSPE) (Avanti Polar Lipids, Alabaster, AL, USA), and cholesterol.

### 2.2. Preparation of Conventional Liposomes and HPLNs

Conventional liposomes were prepared from hydrogenated soy PC, cholesterol, and m-PEG_2000_-DSPE in molar proportions of 57.5 : 37.5 : 5, nontargetable HPLNs prepared from h-PEG_1_PCDA, hydrogenated soy PC, cholesterol, and m-PEG_2000_-DSPE at a molar proportion of 15 : 47 : 32 : 6, and targetable HPLNs prepared from h-PEG_1_PCDA, hydrogenated soy PC, cholesterol, mal-PEG_2000_-DSPE, and m-PEG_2000_-DSPE at a molar proportion of 15 :  47 : 32 : 4.5 : 1.5, and the PLNs prepared from h-PEG_1_-PCDA, m-PEG_750_-PCDA, sulfo-PEG_1_-PCDA and mal-PEG_1500_-PCDA at a molar proportion of 65 : 25 : 5 : 5, according to the method previously described [14]. Briefly, lipids were mixed and evaporated *in vacuo,* to a film. Deionized water or 300 mM ammonium sulfate was added to the films so as to give a 25 mM (total lipid and cholesterol) suspension. The suspension was heated via sonication between 70 and 80°C with a probe-tip sonicator (Fisher sonic dismembrator model 300) for 5 min. The resulting milky solution was then passed through a stacked polycarbonate membrane (100 nm), eleven times, with a dual syringe extruder (LiposoFast-Basic, Avestin, Inc., Ottawa, ON, Canada), heated to 65°C. The nearly clear liposome solutions were cooled to 5°C for 12 hours. After warming to ambient temperature, the water-filled liposomes that contain PCDA lipids were polymerized by UV light irradiation (254 nm) with a Spectrolinker XL-1000 UV Crosslinker (Spectronics Corp.) for 10 minutes. The resulting blue PLNs and HPLNs were heated to 65°C for 5 min to convert them to the red (fluorescent) form. The colored solutions were syringe filtered through 0.2 *μ*m cellulose acetate filters in order to remove trace insoluble contaminants. 

### 2.3. Doxorubicin Loading

The ammonium sulfate-containing conventional and polymerizable (HPLN) liposomes was passed over a G50 Sephadex column (washed with 20 mM HEPES) to exchange the external buffer. The liposomes were then incubated with doxorubicin HCl (Shandong Tianyu Fine Chemical Co., Ltd.) at a concentration of 1 *μ*M of Dox to 3.2 *μ*M of lipid while heating to 65°C for 20 min. The unencapsulated doxorubicin was removed by shaking with anionic exchange resin (Bio-Rex 70, Bio-Rad Inc) in a ratio of 7 *μ*g of doxorubicin to 1 *μ*L of packed resin, for 5 min. Liposomes were separated from resin by filtering through Pierce Spin Cups. The average particle size measurements were obtained on a Zetasizer Nano S (Malvern Inst.), in a solution of 10 mM sodium chloride.

### 2.4. Preparation of ALCAM-Antibody-Conjugated PLNs and HPLNs

An anti-ALCAM antibody was previously engineered into a cys-diabody (cross-paired dimer of single-chain antibody fragments, with C-terminal cysteine residues) as described [19]. PLNs and non-crossslinked, dox-loaded HPLNs, were incubated with anti-ALCAM cys-diabody was conjugated to the particle surface. TCEP (500 mM) that became added to cys-diabody (1–4 *μ*g/*μ*L) solution to a final concentration of 10 mM and incubated at room temperature for 30 minutes to reduce the diabody's terminal cysteine residues. Reduced  *α*-ALCAM cys-diabody was then added to the liposome mixture (2.5–10 *μ*g/*μ*L lipid) at a diabody/lipid ratio of 1 *μ*g diabody : 7.5 *μ*g total lipid and incubated at room temperature for 2 hours to allow for conjugation to maleimide residues on either the PLN or HPLNs. Unbound maleimide residues were quenched with 20 mM cysteine solution for 30 minutes. Unbound diabody, free cysteine, and TCEP were removed using filtration through Amicon Ultra-0.5 mL 100 K Centrifugal Filters (Millipore). Samples were diluted 1 : 2 with HEPES-buffered saline and centrifuged at 6000 rpm for 10 minutes to concentrate the ALCAM diabody conjugated sample. After purification the antibody-conjugated, dox-loaded, non-crosslinked HPLNs were photopolymerized by UV irradiation as described in Section 2.1. The untargeted PLNs were prepared as controls by quenching the maleimide residues with 20 mM cysteine. 

### 2.5. Quantification of Entrapped Liposomal Doxorubicin

Doxorubicin was quantified spectrophotometrically based on the molar extinction coefficient of 12,500. Unencapsulated Dox was removed using Bio-Rex 70. Dox-loaded particles were disrupted using diluted a 1 : 20 isopropanol with 0.075 M HCl solution and then vortexed for at least 30 seconds to ensure complete membrane rupture. Absorbance was read at 480 nm on a Beckman Coulter DU800 spectrophotometer.

### 2.6. Quantification of Total Lipid

Total lipid content of HPLN samples was measured using a colorimetric assay. A 4 *μ*L aliquot of HPLN sample was vacuum dried and resuspended in an ammonium ferrothiocyanate/chloroform solution consisting of 0.1 M Ferric chloride mixed with 0.4 M ammonium thiocyanate and 200 ul of chloroform. Absorbance at 488 nm of the organic phase was then measured in a Beckman Coulter DU800 spectrophotometer. OD488 of the sample was then compared to a standard curve of known lipid concentration values.

### 2.7. Stability of Liposomal Doxorubicin Containment

The ability of nanoparticles (both HPLN and conventional liposome formulation) to hold on to entrapped doxorubicin was measured using a postload time course study. Nanoparticles were loaded with doxorubicin using the procedure previously described. After loading, several conditions of nanoparticle storage were altered to simulate neutral pH of intracellular environment and early and late endosome cellular compartments. Nanoparticles were stored at a pH of 4.5, 6, or 7.4 and at temperature of either 4°C or 37°C giving a total of six different storage conditions. The concentrations of doxorubicin inside the nanoparticles were measured at 0 hr, 0.5 hr, 4 hr, 24 hr, 48 hr, and 144 hr using the method described previously. Before each measurement, any extraliposomal doxorubicin was removed by incubation with BioRex 70 resin (Bio-Rad, Inc.). Each measurement was then normalized to the time-zero measurement of the respective sample to obtain a “percent-contained” doxorubicin measurement in order to assess the stability of the particles. Analysis of variance using log transformed data and blocking on experiment day was used to assess the effect of vehicle, pH, and temperature on doxorubicin leakage, to test for interactions among factors, and to construct 95% confidence intervals for geometric mean fold changes in leakage.

### 2.8. MTT Assay

Osteosarcoma cell lines KHOS 240S, HOS, or MNNG-HOS were grown in Dulbecco's Modified Eagle Medium (HyClone Cat no. SH30022.01) with 10% fetal bovine serum (Gemini Bioproducts). Cells were seeded in a 96-well format at a concentration of 5 × 10^3^ cells/well at a volume of 100 *μ*L media with penicillin/streptomycin and incubated overnight. The following day, wells were treated with doxorubicin-loaded targeted HPLNs, untargeted HPLNs, conventional liposomes, or free doxorubicin for a four-hour period then washed with fresh media. Doses were added based on doxorubicin concentrations ranging on a log scale from 0.01 to 100 *μ*M and at 0 nM. The 0 nM well was treated with HEPES-buffered saline. Each treatment was performed in triplicate. Cells were incubated under standard CO_2_ conditions for 72 hrs at 37°C. At 72 hrs, all wells are treated with 10 *μ*L of thiazolyl blue tetrazolium bromide (Sigma) solution at an initial concentration of 5 *μ*g/*μ*L in phosphate buffered saline and incubated for 4 hrs. Reaction was ceased and cells lysed by adding 100 *μ*L of 15% sodium dodecyl sulfate/15 mM HCl solution and incubated overnight in the dark at room temperature. Plate absorbance was read using Bio-Rad microplate reader at 570 nm. To account for background absorbance, the arithmetic mean of the OD570 of the blank wells was subtracted from the OD570 readings of all treated wells. The arithmetic mean of each plate was calculated and considered as 100% viability. The remaining wells were then divided by this mean to obtain nominal percent viability within each well. Viability was plotted against log drug concentration, and unweighted nonlinear regression was used to estimate log (IC50) for each treatment using a four-parameter sigmoid dose-response model (Prism Software, GraphPad). Fixing the bottom parameter to zero yielded better residual patterns and more stable Hill slope estimates than analyses allowing a variable bottom. For each cell line experiment, a run comparing the four treatment vehicles was repeated 3 to 7 times on different days. Within each cell line, a linear mixed effects model revealed day-to-day variability as a much greater source of variation in log (IC50) than batch variability, and blocking on experiment day improved the precision of estimated differences between treatments. In assessing IC50 results across cell lines, a significant cell line by treatment interaction was detected that could be fully accounted for by modeling a shift in conventional liposome potency (relative to the other 3 treatments) just in the MNNG-HOS cell line.

### 2.9. PLN Binding Fluorescent Microscopy Assay

Osteosarcoma cell lines were seeded onto 4-well Lab-Tek II Chamber Slides (Thermo Scientific) to reach 80% confluence overnight. Cells were treated with anti-ALCAM diabody conjugated PLN at 50 *μ*g/mL per well. Cells were incubated for 4 hrs at 37°C. Media were removed, and wells were washed with 1 mL fresh media. Cell fixation was with 3.7% formaldehyde in Phosphate buffered saline for 15 minutes at 4°C. Cells were mounted using VECTASHIELD mounting medium with DAPI (Vector Laboratories). Positive and negative control cell lines were pancreatic cell lines HPAF and MiaPaca, respectively. Cells were viewed using a Carl Zeiss Axio Imager D1 fluorescence microscope. Cells were viewed at 20x magnification. DAPI was visualized through blue/cyan filter. Bound nanoparticles were visualized using the rhodamine filter at a 1 second exposure.

### 2.10. Western Immunoblot

Antibodies used for immunoblot were monoclonal mouse anti-CD166 (Vector Laboratories, Cat no. VP-C375) at a concentration of 1 : 400 and anti-Actin C-11 (Santa Cruz Biotechnology, Cat no. sc-1615) at a concentration of 1 : 3000.

### 2.11. Immunohistochemistry

Deidentified human patient osteosarcoma paraffin-embedded samples were obtained from the UCLA Tissue Procurement Core Laboratory (IRB Exempt). Four-micrometer sections were cut and placed onto slides. Samples were then deparaffinized, rehydrated, and subjected to heat-induced epitope retrieval. Slides were incubated with a 1 : 50 dilution of anti-CD166 mouse monoclonal antibody (Vector) for 2 h at room temperature, and signal was detected using the mouse EnVision+ System-HRP (DAB) kit (Dako). Sections were counterstained with hematoxylin. Images were viewed and obtained using Zeiss AxioImager at 20x magnification.

## 3. Results

### 3.1. ALCAM Is Highly Expressed in Both Primary Osteosarcoma Specimens and Tumor-Derived Cell Lines

**Figure 1:**
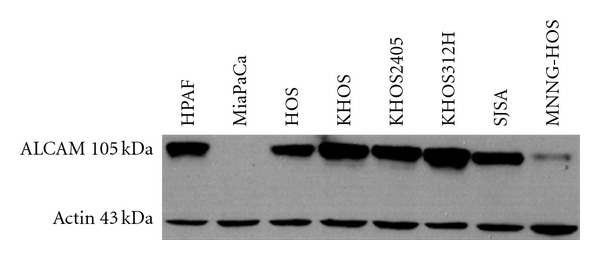
ALCAM/CD166 is highly expressed in osteosarcoma cell lines. Western immunoblot analysis of osteosarcoma cell lines probed with  *α*-ALCAM antisera. There is high total cell expression of ALCAM in HOS, KHOS, KHOS240s, and SJSA with moderate expression in the MNNG-HOS cell line. A pancreatic cancer cell line, HPAF, with known high levels of ALCAM expression was used as a positive control. MiaPaCa, a pancreatic cancer cell line with known lack of ALCAM expression served as a negative control. Membrane-localized ALCAM isoform is present at 105 kDa. *β*-Actin was used as an internal loading control.

**Figure 2:**
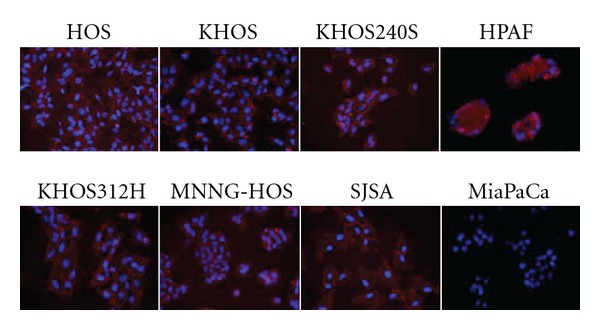
Surface ALCAM expression in osteosarcoma cell lines. Fluorescent immunohistochemistry showing membranous ALCAM expression in osteosarcoma cell lines in comparison to a known ALCAM expressing pancreatic cancer cell line, HPAF (positive control), and ALCAM negative cell line, MiaPaCa. ALCAM binding shown in red (Alexa Fluor 564) and nuclei counterstained with DAPI (20x magnification).

 A molecular survey of the osteosarcoma cell line U2-OS demonstrated expression of ALCAM on the surface of these cells [15]. These observations prompted a more in-depth investigation of ALCAM expression in human osteosarcoma. Evaluation of ALCAM expression in a collection of 6 tumor-derived cell lines was used as an initial platform. Cell lysates were harvested from subconfluent adherent cultures grown in tissue culture and analyzed by immunoblot using anti-ALCAM antisera. Pancreatic cancer cell lines with high (HPAF) and no (MiaPaCa) ALCAM expression were used as controls. All 6 osteosarcoma cell lines expressed ALCAM, and 5 of 6 demonstrated elevated expression at the level seen in the HPAF control (Figure 1). The quality of ALCAM expression was further confirmed in fluorescent immunohistochemistry showing primarily a membranous, surface component to the ALCAM expression in osteosarcoma cell lines (Figure 2).

 Though there was a high frequency of ALCAM expression in our cell line collection, there is always a concern that it may be due to a selection process inherent in creating tumor-derived cell lines. In addition, differences in growth conditions between in vivo in osteosarcoma patients and in vitro in tissue culture may be responsible for changes in ALCAM expression. 

**Figure 3:**
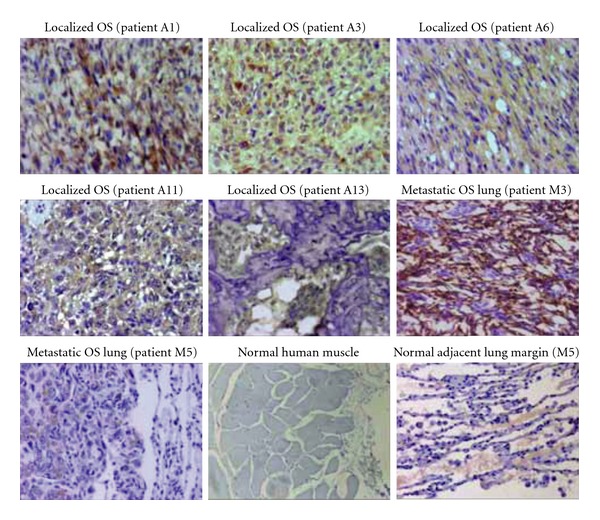
ALCAM expression in human osteosarcoma patient tumor samples. Immunohistochemistry studies in human paraffin-embedded localized and pulmonary metastatic osteosarcoma samples show mild to strong expression of ALCAM (brown) both membranous and cytoplasmic in appearance in comparison to normal human muscle and human lung. Tissues were counterstained with hematoxylin (magnification 20x).

To address these concerns, human osteosarcoma tumor samples both from primary and metastatic sites were assayed for ALCAM expression by immunocytochemistry. Banked anonymized patient specimens were fixed, sectioned, and incubated with anti-ALCAM antisera. After washing, in situ ALCAM expression was detected using a colorimetric assay and evaluated by light microscopy. Tissues were graded as strongly positive (+++), moderately positive (++), weakly positive (+), or negative (−). All OS tumor samples stained positively for ALCAM. Of 10 localized and metastatic OS samples, 5 of the localized OS tissues stained weakly to strongly positive for ALCAM and 5 of the metastatic OS samples also had moderate to strong IHC staining. Osteosarcoma cells demonstrated both cytoplasmic and membranous ALCAM expression (representative IHC images are shown in Figure 3). 

### 3.2. Anti-ALCAM Coupled Polymerized Liposomal Nanoparticles Avidly Bind to Osteosarcoma Cell Lines

 Polymerized liposomes and hybrid polymerized liposomal nanoparticles (PLNs and HPLNs) were evaluated as a potential therapeutic delivery vehicle that could be targeted to osteosarcoma cells expressing ALCAM. PLNs and HPLNs share many structural attributes of conventional liposomes. They are self-assembling unilamellar spheres whose surfaces can be modified using the same chemical coupling strategies as employed for liposomes. Unlike liposomes, PLNs and HPLNs can be manufactured to be intrinsically fluorescent. Ultraviolet irradiation leads to cross-linking of diacetylene residues present in their acyl chains, leading to highly colored blue particles, and heat treatment of the PLNs and HPLN vesicles leads to color change and fluorophore formation [16, 17]. The fluorescence emission spectrum is centered at 635 nm with a broad and complex excitation spectrum from 480 to 580 nm. As a result, PLNs and HPLNs converted into their fluorescent form can be readily traced from the time they bind to target cells until they are deposited and compartmentalized into subcellular structures.

 Targeted PLNs and HPLNs were created by chemically coupling a recombinant anti-ALCAM antibody fragment to its surface. PLNs and HPLNs were synthesized containing maleimide reactive groups at the distal end of surface polyethylene glycol (PEG) molecules. A bivalent anti-ALCAM diabody, derived from a previously described scFv [18], was genetically engineered to contain a C-terminal cysteine [19]. The resulting cys-diabody is bivalent, compact (one-third the size of an intact antibody) and enables site-specific oriented coupling of the antibody variable regions to the surface of nanoparticles [20]. Mixing these two components induced a condensation reaction between the thiol of the cysteine and the maleimide moiety, resulting in the anti-ALCAM diabody being covalently coupled to the PLN or HPLN surface. As a negative control, untargeted HPLNs where prepared without maleimide lipid and untargeted PLNs were made by coupling free cysteine to nanoparticles.

**Figure 4:**
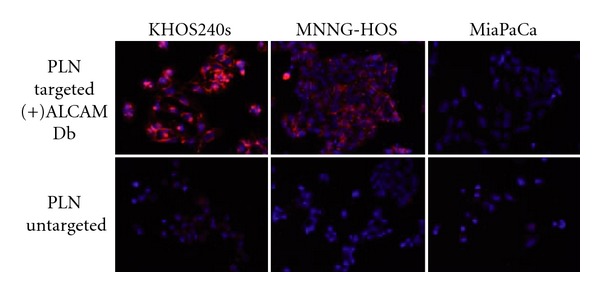
*α*-ALCAM-targeted PLNs bind specifically to osteosarcoma cell lines.  *α*-ALCAM cys-diabody conjugated PLNs show specific binding (red) to two osteosarcoma cell lines expressing ALCAM, KHOS240s and MNNG-HOS. There was no binding to a cell line that does not express ALCAM, MiaPaCa. Fluorescence microscopy of PLNs is shown in red and DAPI nuclear counterstaining in blue (magnification 20x).

 Binding studies were performed comparing the relative affinities of anti-ALCAM coupled PLNs (*α*-AL-PLN) versus untargeted PLNs towards osteosarcoma cell lines. After a 4-hour incubation, cells were washed and (*α*-AL-PLN) binding was detected by fluorescence microscopy.  *α*-AL-PLNs bound to all of the osteosarcoma cell lines in our panel, much more efficiently than untargeted negative controls (Figure 4). This interaction was dependent on cellular ALCAM expression. Both targeted and untargeted PLNs bound equally to MiaPaCa cells that do not express cell surface ALCAM.

 To gauge the rapidity of the interaction between  *α*-AL-PLNs and osteosarcoma cells, a time course study was performed. Osteosarcoma cells were incubated with  *α*-AL-PLNs for varying time periods up to 4 hours, washed, and then evaluated by fluorescence microscopy. 

**Figure 5:**
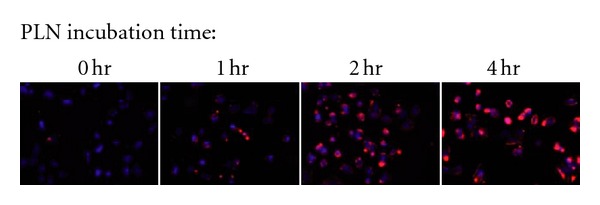
*α*-ALCAM-conjugated PLNs show cell-specific targeting in a time-dependent manner.  *α*-ALCAM-conjugated PLNs were incubated with osteosarcoma cells for 0, 1, 2, and 4 hours then washed off.  *α*-ALCAM targeted PLNs bind specifically to the ALCAM expressing osteosarcoma cell line KHOS240s at one hour and reach a maximum binding at 4 hours of incubation. Fluorescence microscopy of PLNs (shown in red) with DAPI (blue) nuclear counterstaining (magnification 20x).

**Figure 6:**
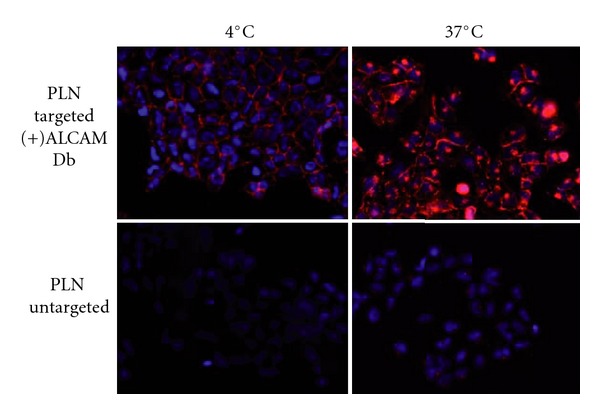
*α*-ALCAM-conjugated PLNs are internalized via receptor-mediated endocytosis.  *α*-ALCAM-targeted PLNs incubated with the osteosarcoma cell line KHOS240s at 4 degrees and at 37 degrees Celsius show inhibition of receptor-mediated endocytosis at 4°C and rapid internalization of targeted PLNs at 37°C versus untargeted PLNs that do not bind under either circumstance. Fluorescence microscopy of PLNs (shown in red) with DAPI (blue) nuclear counterstaining (magnification 20x).

*α*-AL-PLNs binding was detected as early as 30 minutes and reached a maximum by 4 hours (Figure 5). The presence of a strong perinuclear fluorescence signal suggested that the targeted nanoparticles were rapidly internalized into the endosome compartment of the cell. To further evaluate this, binding studies were performed at 4°C, which would inhibit cellular endocytosis. Under these conditions, a strong membrane fluorescence signal was detected without perinuclear nuclear localization consistent with  *α*-AL-PLNs being bound to the cell surface but not internalized (Figure 6).

### 3.3. Hybrid PLNs Were Formulated to Function as Potential Therapeutic Delivery Vehicles

 Our initial PLN formulation was composed entirely of 10,12-pentacosadiynoic acid (PCDA) derivatives and when polymerized formed a very fluorescent particle that could easily be detected. However, these nanoparticles proved to be problematic when trying to adapt them for delivery of therapeutics. Attempts at effectively loading them with cytotoxic chemotherapeutic agents, either through encapsulation during vesicle formation or across ion gradients using the prepolymerized liposomes, failed at multiple levels. For this reason, hybrid PLNs were created which composed of PCDA lipids mixed with saturated phospholipids found in many liposome formulations. 

 To approach this problem, we started with a standard liposomal formulation consisting of hydrogenated soy PC (where the major component is distearoylphosphatidylcholine (DSPC)), cholesterol, and polyethylene glycol-distearoylphosphatidyl ethanolamine (m-PEG_2000_-DSPE) in molar proportions of 57.5 : 37.5 : 5. Increasing amounts of unsaturated PCDA lipids were then added. We chose a very short PEG chain PCDA derivative, h-PEG_1_-PCDA, because it is an extremely reactive cross-linking lipid, has good aqueous dispersion properties when mixed with charged lipids, and is in itself uncharged so it will not alter the overall surface charge, and the small polar head will not interfere with the conjugation of targeting agents. After sonication and extrusion, vesicles were evaluated for size by dynamic light scattering and the ability to form fluorescent particles when treated with UV irradiation and heat. After overnight cooling to 5°C, we found that inclusion of as little as 15 mole% h-PEG_1_-PCDA resulted in brightly blue-colored particles but that this color became progressively attenuated with decreasing h-PEG_1_-PCDA proportions.

 Considering that our HPLNs were a heterogeneous mix of lipids with two very different acyl chain structures, stability in solution was a major concern. Certain hybrid formulations formed insoluble aggregates within hours after extrusion. Overnight cooling at 5°C immediately after extrusion, but prior to polymerization, proved to be critical in creating stable HPLNs. Particles treated this way were stable for weeks either refrigerated or at room temperature. Hybrid PLNs of the same lipid composition constructed without this cooling step were irretrievably unstable. 

 From these studies, an optimized HPLN formulation was empirically derived consisting of h-PEG_1_-PCDA, hydrogenated soy phosphatidylcholine, DSPC, cholesterol, and m-PEG_2000_-DSPE at a molar proportion of 15 : 47 : 32 : 6. Using this formulation, HPLNs were fabricated and their ability to be actively loaded with doxorubicin through generation of an ion gradient was assessed [21]. Using this method, doxorubicin could be loaded into HPLNs to an average final drug/lipid molar ratio of 0.15 (range 0.13–0.18) in comparison to conventional PEG-liposomes lacking PCDA lipids which could be loaded to an average molar ratio of 0.44 (range 0.35 to 0.49). 

**Figure 7:**
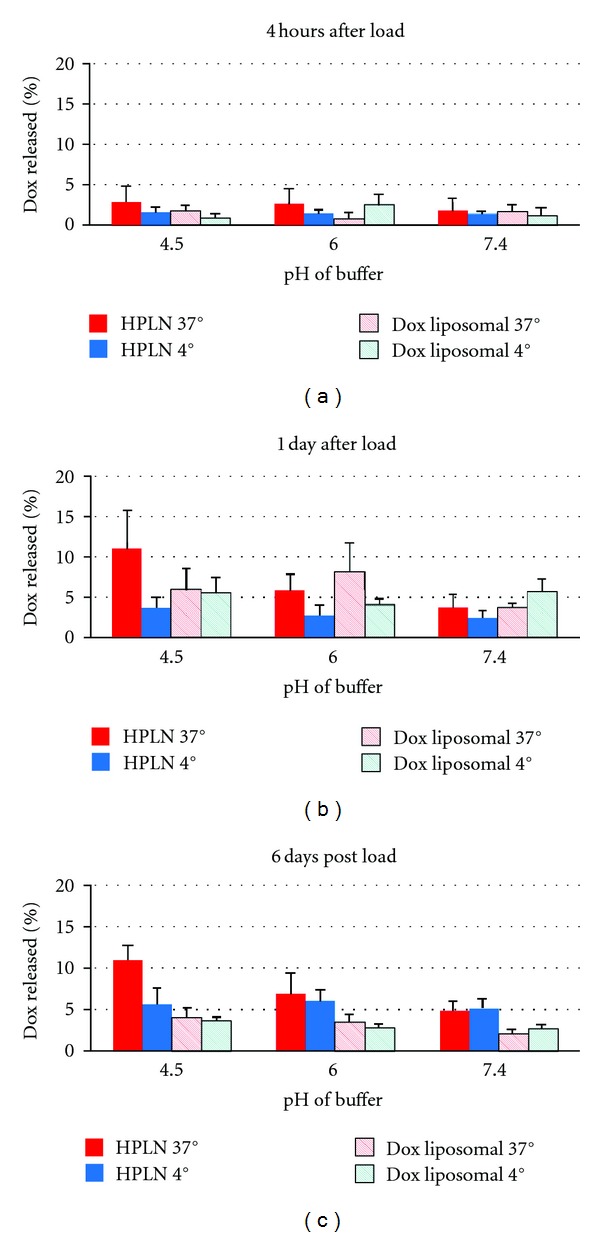
Containment of doxorubicin over time in drug-loaded liposomal vehicles. Containment studies of loaded HPLNs versus conventional PEG-liposomes showed that leakage increased significantly with time (*P* < 0.0001), with geometric means at 4 hours, 1 day, and 6 days being, respectively, 0.9%, 4.6%, and 3.1% for conventional doxorubicin-loaded PEG-liposomes and 1.2%, 3.2%, and 5.9% for HPLN. There were no significant differences between the vehicles or the effects of pH or temperature that could be detected at 4 hours or 1 day. However, at 6 days HPLN had 1.9-fold greater overall leakage than DOX (*P* < 0.001; 95% C.I. 1.6- to 2.4-fold). Lowering pH from 7.4 to 4.5 increased leakage by a factor of 1.5 (*P* = 0.01; 95% C.I. 1.1- to 2.1-fold) with enhanced leakage at 37 degrees compared to 4 degrees (*P* = 0.03).

 Containment studies of loaded HPLNs versus conventional PEG-liposomes showed that leakage increased significantly with time (*P* < 0.0001), with geometric means at 4 hours, 1 day, and 6 days being, respectively, 0.9%, 4.6%, and 3.1% for conventional doxorubicin-loaded PEG-liposomes and 1.2%, 3.2%, and 5.9% for HPLN (Figure 7). No significant differences between the liposomal vehicles or the effects of pH or temperature could be detected at 4 hours or 1 day. However, at 6 days at 37 degrees HPLN had 1.9-fold greater overall leakage than DOX liposome at either 4 or 37 degrees (*P* < 0.001; 95% C.I. 1.6- to 2.4-fold). In addition, lowering pH from 7.4 to 4.5 increased drug release in HPLN by a factor of 1.5 (*P* = 0.01; 95% C.I. 1.1- to 2.1-fold) with evidence that this effect was enhanced at 37 degrees compared to 4 degrees (*P* = 0.03).

### 3.4. Untargeted Doxorubicin-Loaded HPLNs Are More Cytotoxic to Osteosarcoma Cells Than Liposomal Doxorubicin Formulations

 Since doxorubicin is a mainstay in the current treatment of osteosarcoma, it was chosen as our initial payload to test whether HPLNs could serve as therapeutic delivery vehicles. HPLNs and conventional liposomes were fabricated by the same procedure of hydration of dried lipid films by brief sonication followed by extrusion through 100 nm polycarbonate filters. The sizes of HPLNs and liposomes were approximately the same varying from batch to batch from 90 to 110 nm with a typical polydispersity index of about 0.1. Both particles were loaded with doxorubicin using ammonium sulfate gradients. Prior to dosing cells, loaded nanoparticles were incubated briefly with an anionic exchange resin (BioRex 70, BioRad Inc) to scavenge any nonencapsulated (free) doxorubicin. This ensured that cells were not exposed to free drug that may have leaked out while particles were in storage. Nonconfluent osteosarcoma cell lines were then incubated for 4 hours with varying concentrations of doxorubicin-loaded HPLNs or liposomes in triplicate. Cells exposed to free doxorubicin (DOX) served as positive controls. After dosing, cells were washed with fresh media and incubated for a total of 72 hours. Cell viability was then quantified by MTT assay, and 50% inhibitory concentrations (IC50s) were estimated. For each osteosarcoma cell line, this experiment was performed 3–7 times using at least two different batches of HPLNs and liposomes.

**Figure 8:**
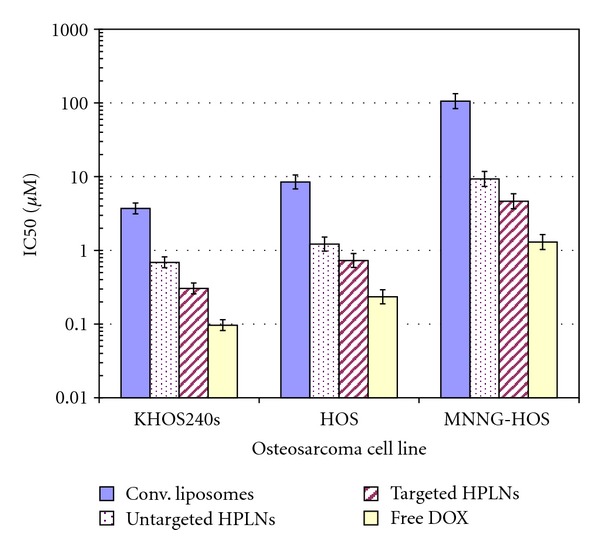
Cytotoxicity IC50s for doxorubicin-loaded vehicles and free DOX. Targeted and untargeted hybrid PLNs were incubated with osteosarcoma cell lines in comparison to free doxorubicin and conventional PEG-liposomal doxorubicin. Mean log IC50 for osteosarcoma cell lines shows that untargeted hybrid PLNs have a 6-fold increase in cytotoxicity over conventional liposomal formulation. ALCAM targeted hybrid PLNs (HPLN) show an additional 2-fold increase over untargeted HPLNS and 12-fold increase in cytotoxicity over conventional PEG-liposomal doxorubicin. Geometric mean IC50s were derived from seven, five, and three sets of MTT assay experiments in, respectively, the KHOS240s, HOS, and MNNG-HOS cell lines. Error bars correspond to 1 standard error for mean log (IC50). Within each cell line, differences among vehicles are highly significant (*P* < 0.0001). Confidence intervals for pairwise ratios of IC50s, corrected for multiple comparisons, are summarized in Table 1.

 Absolute IC50 values for each doxorubicin preparation varied according to osteosarcoma cell line (Figure 8). However, the trend reflecting the relative potency of these preparations was consistent across all cell lines tested. As has been seen previously in other cell models, free doxorubicin was approximately 38- to 82-fold more potent than conventional liposomal doxorubicin [22]. Loaded HPLNs without targeting (HPLN/Dox) showed intermediate potency that was about 6-fold greater than the conventional PEGylated liposomal preparation.

 Follow-up experiments were performed to determine whether the increased growth inhibition mediated by loaded HPLN/Dox was related to the amount of PCDA lipid in this formulation. HPLN/Dox with reduced PCDA were fabricated, loaded, and incubated with the KHOS240S osteosarcoma cell line. Though these variant HPLN/Dox formulations were of similar size and loaded equally well with doxorubicin, decreasing the PCDA lipid composition resulted in nanoparticles with decreased growth inhibitory potency (data not shown).

### 3.5. ALCAM Targeting Enhances the Growth Inhibitory Effect of Doxorubicin-Loaded PLNs

 We have shown that coupling anti-ALCAM diabodies to the surface of PLNs increases their binding affinity for osteosarcoma cell lines (Figure 4). This same effect was found using  *α*-AL-HPLNs (data not shown). Experiments were then performed to determine whether this targeting function improved the ability of doxorubicin-loaded HPLNs to inhibit growth of osteosarcoma cell lines. Targetable HPLNs were fabricated using h-PEG_1_-PCDA, hydrogenated soy phosphatidylcholine, cholesterol, Mal-PEG_2000_-DSPE, and m-PEG_2000_-DSPE at a molar ratio of 15 : 47 : 32 : 4.5 : 1.5. The proportion of maleimide DSPE was empirically determined as the lowest amount that when coupled to anti-ALCAM diabody would result in enhanced binding to osteosarcoma cells (data not shown). After loading with doxorubicin, HPLNs were coupled to anti-ALCAM diabody as before giving  *α*-AL-HPLN/Dox. Osteosarcoma cells were then incubated with  *α*-AL-HPLN/Dox as previously described.

 As seen with untargeted hybrids, the absolute sensitivity to  *α*-AL-HPLN/Dox varied across different osteosarcoma cell lines (Table 1). However, in all cases, the targeted HPLNs demonstrated an additional growth inhibitory potency over untargeted HPLN counterparts of approximately 2-fold in all cell lines (Figure 8). Taken together,  *α*-AL-HPLN/Dox had a log order (12-fold) increase in cytotoxicity over the conventional untargeted PEG-liposomal doxorubicin formulation in KHOS240s and HOS cell lines while having a 23-fold increase in the chemoresistant MNNG-HOS cell line. This implies that  *α*-AL-HPLN/Dox can both specifically bind cells and deliver doxorubicin to achieve greater cytotoxicity over a conventional untargeted liposomal nanoparticle formulation.

## 4. Discussion

 Our data clearly demonstrate an increase in ALCAM expression in osteosarcoma though the biologic consequences of this are difficult to gauge. The normal physiologic roles of ALCAM are still coming to light, but its molecular structure and clustering at tight junctions suggest that it could be involved in cell adhesion and migration [23]. In this context, it is tempting to think that modulating ALCAM expression could potentiate the invasive and metastatic behaviors found in high-grade malignancies such as osteosarcoma. However, there is no consistent correlation between ALCAM expression level and patient survival across all cancers. For example, an increase in ALCAM expression is found in higher-stage more aggressive malignant melanoma [24]. By contrast, high ALCAM is correlated with low-grade less aggressive cases of prostate cancer [11]. Considering the high frequency of elevated ALCAM expression in even our small cohort of osteosarcomas, it may not be able to discriminate between high- and low-risk patients with this disease.

 Though ALCAM may be a limited prognostic biomarker in osteosarcoma, it has potential to serve as a molecule through which to therapeutically target this tumor. Fluorescent nanoparticles coated with anti-ALCAM diabodies preferentially bind to osteosarcoma cell lines, even those that express ALCAM at relatively low levels. As seen in prostate cancer cells, ALCAM-targeted nanoparticles were rapidly internalized by osteosarcoma cells suggesting a strategy for intracellular delivery of anticancer agents.

 The use of diacetylene containing lipids to create polymerizable films and vesicles has been intensively studied for creating biosensors [25] and have been explored as cancer diagnostic and delivery vehicles [26]. When these membranes are treated with ultraviolet irradiation the resulting intralipid cross-links form an intensely blue chromophore. When exposed to physiochemical perturbations such as heat, shear, or pH stress, these membranes shift from a blue nonfluorescent state to a red fluorescent state [17]. A distinct advantage to the HPLN fluorescence is that little or no photobleaching occurs. Taking advantage of these properties, we were able to track binding and internalization of red, fluorescent ALCAM-targeted PLNs (*α*-AL-PLN) that had been treated with UV irradiation and heat. Interestingly, we obtained the same results using a similar preparation of  *α*-AL-PLN that received only UV irradiation and were therefore blue and nonfluorescent in solution (data not shown). It appears that the interaction between the coupled diabody molecules and the cell surface ALCAM proteins exerted sufficient stress to shift the bound  *α*-AL-PLN into a fluorescent state.

 Though vesicles composed entirely of diacetylene containing lipids had excellent detection properties, they had limited capability as therapeutic delivery vehicles. We were unable to stably load these liposomes with doxorubicin either by passive encapsulation during vesicle formation or actively across ion gradients in formed vesicles. Others have been able to passively load hybrid liposomes composed of a 1 : 1 mixture of a phosphatidylcholine derivative with a dichain diacetylene lipid and another phospholipid [27]. However, loading efficiencies were low and this strategy may be limited to hydrophobic payloads. We have found that for amphiphilic molecules such as doxorubicin, in HPLNs, with single-chain, neutral PCDA lipids the polymerizable lipid concentration needs to be 20 mole percent or less for efficient loading to occur (data not shown).

 Though our HPLNs were initially formulated for their stable drug loading characteristics, they surprisingly also proved to be more therapeutically potent in in vitro testing. The IC50 concentrations of untargeted doxorubicin-loaded hybrid PLNs in three independent osteosarcoma tumor-derived cell lines were at least 6-fold lower than conventional liposomal doxorubicin composed of PEGylated saturated phospholipid. This boost in potency appears to depend on PCDA lipid content since it is progressively lost as the PCDA concentration is titrated down from an optimum of 15–20 mole percent (data not shown). From this point, the lower the PCDA lipid concentration is in our HPLNs, the higher the IC50 becomes in our osteosarcoma model. Recently, others have used mixtures of diacetylene lipids and phospholipids to create liposomes that could be selectively destabilized either by photochemical means or by thermal shock [28, 29]. The goal here was to create a therapeutic vehicle that would release its payload in a temporal spatially controlled fashion. 

We have found that even without applying an external destabilizing stimulus, HPLNs can be more effective therapeutic delivery vehicles than standard liposomal formulations. The mechanisms underlying this effect are unclear and require further investigation. The presence of PCDA in our hybrid formulations could be having an effect at multiple steps in our in vitro assay from (i) nanoparticle binding to cells to (ii) cellular uptake to (iii) intracellular release of cytotoxic payload. This last step in particular may be rate limiting. The roughly 50-fold difference in IC50 between free doxorubicin and conventional liposomal doxorubicin seen in our osteosarcoma cell lines is consistent with that found in previously published model systems [22]. Others have shown that this is primarily due to delayed release of free drug from the endocytic compartment of cells that have taken up liposomal doxorubicin [30].

Evaluating the stability of doxorubicin drug containment within the HPLN versus conventional PEG-liposomes showed that there was a statistically significant increase in doxorubicin release from the HPLN over time. Furthermore, this enhanced drug release was accentuated under acidic conditions mimicking the receptor-mediated endocytic environmental conditions of late endosomes and lysosomes. It is tempting to hypothesize that the PCDA lipids may enhance the release of doxorubicin from HPLNs that have been taken up by osteosarcoma cells. Given their differences in molecular structure, it is highly likely that microsegregation occurs between PCDA lipids and phospholipid molecules on the surface of HPLNs. Evidence found in published studies with similar mixtures of longer chain diacetylene lipids and shorter chain phosphatidylcholine lipids suggests that a phase separation occurs between the lipid types [31]. It is possible that these PCDA lipid islands could serve as destabilization points that could enhance drug release when exposed to intracellular environments. 

The creation of an osteosarcoma-targeted doxorubicin loaded HPLN (*α*-AL-HPLN/Dox) resulted in a 2-fold increase in cytotoxicity over the untargeted HPLN/Dox and a 12-fold increase in cytotoxicity over the conventional PEG-liposomal formulation in the HOS and KHOS240s osteosarcoma cell lines. These results suggest that ALCAM targeting in osteosarcoma adds an incremental therapeutic effect. Interestingly, in the MNNG-HOS cell line the  *α*-AL-HPLN/Dox had an even greater increase (23-fold) in cytotoxicity over the PEG-liposomal formulation. The MNNG-HOS cell line has high expression levels of the multidrug resistant protein 1 (MDR1) conferring chemotherapeutic resistance to doxorubicin [32]. The increased sensitivity of the MNNG-HOS chemoresistant cell line to the  *α*-AL-HPLN/Dox formulation over the conventional formulation points to a therapeutic effect that may overcome multidrug resistance. We can hypothesize that the targeting and improved sustained drug release characteristics of our  *α*-AL-HPLN/Dox formulation may help to bypass or overwhelm the drug efflux proteins mediating chemoresistance thereby improving cytotoxicity. 

In conclusion, we have found a novel surface marker in human osteosarcoma, ALCAM, which we have used to specifically target osteosarcoma cells with a novel engineered drug-loaded hybrid PLN formulation anti-ALCAM immunoconjugate. These  *α*-AL-HPLN/Dox particles show improved cytotoxicity over a conventional untargeted PEG-liposomal doxorubicin formulation and show promise as a potential therapeutic delivery platform in osteosarcoma. This new liposomal nanoparticle formulation is particularly attractive for its potential therapeutic application in resistant, refractory, and metastatic osteosarcoma where current standard systemic untargeted chemotherapy is generally not efficacious and prognosis is dismal. Furthermore, the bystander and dose-limiting side effects of systemic chemotherapy are substantial. Thus far this formulation has only been tested in tissue culture based assays, so further assessment in tumorigenic animal models is a crucial next step to validate these findings. These experiments are currently under way.

## Figures and Tables

**Table 1:** Relative cytotoxic potency of doxorubicin-loaded vehicles: ratio of (IC50)^−1^ between vehicles summarized by geometric mean (95% confidence interval)^a^.

Osteosarcoma cell line	Untargeted HPLNs	Targeted HPLNs	Targeted HPLNs	Free Dox
	over	over	over	over
	conventional liposomes	untargeted HPLNs	conventional liposomes	targeted HPLNs
KHOS240s	5.4 (3.7–8.0)	2.3 (1.5–3.3)	12.2 (8.3–18.0)	3.1 (2.1–4.6)
HOS	7.0 (4.8–10.1)	1.7 (1.1–2.4)	11.6 (8.0–16.8)	3.1 (2.1–4.5)
MNNG-HOS	11.4 (5.4–23.9)	2.0 (0.95–4.2)	22.8 (10.8–47.8)	3.6 (1.7–7.5)

Summary of all 3 lines^b, c^	5.9^c^ (4.6–7.6)	2.0 (1.6–2.5)	11.8^c^ (9.2–15.2)	3.2 (2.6–4.1)

^a^Tukey HSD 95% confidence intervals for multiple comparisons in each cell line.

^b^Based on linear mixed effects model for log IC50 as a function of cell line and vehicle.

^c^Indicated potency ratio is 2.0-fold greater (95% CI: 1.4–2.8) in MNNG-HOS cell line, consistent with significant cell line by vehicle interaction (*P* = 0.015).
